# Environmental Impacts Assessment in Suspension PVC Production Process Using Computer-Aided Process Engineering

**DOI:** 10.3390/polym15132902

**Published:** 2023-06-30

**Authors:** Ángel Darío González-Delgado, Miguel Ramos-Olmos, Eduardo Aguilar-Vásquez

**Affiliations:** 1 Nanomaterials and Computer Aided Process Engineering Research Group (NIPAC), Chemical Engineering Department, Universidad de Cartagena, Cartagena, Bolivar, Colombia; eaguilarv@unicartagena.edu.co; 2 Grupo de Investigación en Ciencias Administrativas y Seguridad y Salud en el Trabajo (CIASST), Business Administration Department, Universidad Minuto de Dios-UniMinuto, Cartagena, Bolívar, Colombia; miguel.ramos.o@uniminuto.edu

**Keywords:** polymers, sustainability, PVC, environmental analysis, computer-aided process engineering

## Abstract

The new demands for sustainable operation in the chemical industry due to increasing environmental regulations and agreements have generated the need to adapt existing processes to more intelligent production. The plastics sector is in a complex position due to its contribution to economic development and the climate crisis. Therefore, environmental assessment has become an important tool due to the benefits it provides by quantifying the environmental performance of processes, allowing it to balance operational and environmental needs. Polyvinyl chloride (PVC) is one of the most globally used polymers thanks to its resistance, flexibility, and cost-effectiveness. The polymer is synthetized by suspension polymerization, which is characterized by high productivity and controllability. However, it presents problems associated with intensive energy consumption and the emission of toxic substances and greenhouse gases. Therefore, an environmental assessment of the suspension PVC production process was performed using the waste reduction algorithm (WAR). The potential environmental impact (PEI) was quantified using the generation rate and the output velocity for four cases and three different fuels. It was found that the process transforms raw materials with high impacts, such as VCM, into substances with lower PEI, such as PVC. However, the process has a high generation of PEI due to the effects of energy consumption (−2860, −2410, 3020, and 3410 for cases 1–4, respectively). The evaluation of the toxicological impacts shows that the ATP category is the only one that presents a positive generation value (75 PEI/day); the product contributes to the formation and emission of impacts. The atmospheric categories showed that the energy consumption of the process is the most critical aspect with a contribution of 91% of the total impacts emitted. The AP and GWP categories presented the highest values. It was determined that the most suitable fuel is natural gas; it has lower impacts than liquid and solid fuels (coal). Additionally, it can be concluded that the PVC production process by suspension is environmentally acceptable compared to the polyethylene or polypropylene processes, with output impacts 228 and 2561 times lower, respectively.

## 1. Introduction

Plastics are a key material in people’s daily lives, greatly benefiting human development due to their endurance, flexibility, and low price. However, the manufacturing, usage, and disposal management of plastics have raised environmental concerns ranging from environmental persistence, marine debris, health risks, and greenhouse gas emissions, among others [1]. Polyvinyl chloride is among the world’s top three most utilized polymers [2]. This low-cost polymer has properties such as easy film formation, high processability, excellent biochemical resistance, thermal stability, adhesive capabilities, rigidity, and high compatibility with multiple additives, among others [3]. Its uses are mainly in the building, electricity, food, and health sectors [4]. Approximately 80% of the PVC produced worldwide is obtained through the suspension polymerization method [5], which is known for its high output capacity, ability to create flexible polymer compositions, high level of control, and cost-effectiveness [6]. However, the PVC value chain shows severe environmental problems in the production, consumption, use, and final disposal stages. The problems in the production stage are associated with intensive energy consumption, the emission of toxic substances, risks to human health, the emission of greenhouse gases, and low reuse, among others [7].

Sustainability has become fundamental to the competitiveness of production companies due to the restrictions that new regulations and international agreements place on process operations [8]. The application of environmental impact quantification techniques or methodologies has grown in industry and academia due to their benefits in achieving sustainable operations [9]. This quantification is achieved through computer-aided process engineering (CAPE). This multidisciplinary tool eases the management of the high number of variables, parameters, and systems that form chemical processes, and it is the basis for process assessment such as the waste reduction (WAR) algorithm and its graphical user interface (GUI) [10]. The WAR GUI software is a straightforward tool developed by the Environmental Protection Agency of the United States (EPA) to assess the environmental impacts of chemical processes. It considers only the manufacturing stage of a product’s entire life cycle, which minimizes the information required to conduct the evaluation [11].

Several investigations have been carried out on processes in a wide range of areas and stages of chemical process development using the WAR GUI software. Studies have applied this tool to emerging technologies such as biomass processing. Moreno-Sader et al. used the WAR algorithm and the TRACI tool to evaluate chitosan production from shrimp exoskeletons [12]. Gonzalez-Delgado et al. assessed the environmental performance of a mead production process using the WAR algorithm at a pilot scale and through a simulation (Aspen Plus) [13]. On the other hand, this tool has been applied to proven technologies such as those which use fossil-derived compounds as raw materials or products. There are studies, such as that of Cardona et al., where the WAR algorithm was used to compare the environmental performance of two butyl acetate production processes (conventional and reactive distillation) [14]. Gonzalez-Delgado et al. used the WAR algorithm to assess the environmental performance of a natural gas treatment unit for sulfide and mercaptan removal [15]. 

Additionally, the WAR GUI software can complement other types of process evaluations, such as economic, exergetic, and social processes, among others. Works such as Meramo-Hurtado et al. combined exergy and environmental analyses of chitosan production from shrimp exoskeletons using the WAR algorithm. AspenPlus simulation provided mass and energy data [16]. Duarte et al. employed the WAR algorithm together with economic and social analysis to evaluate the sustainability of a bioethanol production process from coffee waste. Aspen Plus provided technical data (mass and energy flows) [17].

The limited number of studies about the environmental impacts of PVC are concentrated on the use of life cycle analysis (LCA) in combination with CAPE tools such as ReCiPe, and TRACI, among others. These studies examine the PVC resin production as it is carried out in this work but expand this examination to other stages of the PVC value chain such as raw material production, the manufacturing of final products, and other stages. Investigations such as Comanita et al. quantified the environmental impacts of the PVC process, considering the PVC and VCM production [18]. Ye et al. studied the environmental impacts of virgin (s-PVC) and recycled PVC production (r-PVC). The VCM and the end-product stages were considered. Franklin Associates quantified the environmental impacts of the s-PVC manufacturing process, considering both the polymer production process and raw materials (VCM, chlorine, and ethylene) (cradle-to-gate) using operational data from actual chemical plants [19]. These works found the presence of multiple significant environmental issues beyond the sole PVC resin production process, the emissions of highly toxic substances to the environment and human health, and the critical role of intensive energy consumption and its atmospheric contamination throughout the whole value chain [20]. 

Nonetheless, these analyses are limited to the quantitative aspect of the environmental assessment but have no means to evaluate the velocity (rate) of these emitted impacts and their effects on a specific environment. According to the literature research undertaken by the authors, no studies have assessed only the environmental performance of PVC resin production by suspension; earlier works assessed the whole PVC chain and showed that the productive chain of this plastic has serious environmental impacts. For these reasons, this research aims to assess the environmental performance of the suspension PVC production process with unreacted monomer recovery and PVC drying stages using CAPE. The WAR GUI software was used since it considers the generation and output rate of potential environmental impacts (per unit of product produced and per unit of time) and quantifies the energy consumption and product flow contribution to these impacts.

## 2. Materials and Methods

### 2.1. Environmental Assessment Using WAR Algorithm

The WAR Algorithm is a system based on metrics or indicators that allow the quantification of the potential environmental impact caused by the manufacture of products from chemical industry operations and indicates the rate at which the process could have an environmental impact [21]. Estimating the output and the generation of potential environmental impacts cannot be performed by other methods, such as basic life cycle analysis [22]. The software introduces the idea of potential environmental impact and assesses it across eight distinct impact categories, which are categorized into two groups: toxicological and atmospheric impacts.

The toxicological impacts are divided into four categories, the first of which is human toxicity potential by ingestion (HTPI), which estimates the toxicity of a chemical compound. It is calculated for chemical species in liquid or solid state at a temperature of 273 K and a pressure of 1 atm [23], and it is defined by Equation (1):

(1) $$HTPI=\frac{1}{{LD}_{50}}$$

HTPI is also defined as the inverse of the lethal dose, which is the amount of substance that produces death in 50% of rats by oral ingestion of the chemical specie (LD_50_). This indicator is quantified in units of mg of chemical product per kg of rat.

The second category is the human toxicity potential by inhalation or dermal exposure (HTPE). To estimate the HTPE, the threshold limit values (TLV), which are averaged over an 8-h period, are utilized [23]. These values are consigned in the OSHA, ACGIH, and NIOSH databases and express the limit values of exposure for substances that threaten human health by inhalation and/or dermic exposition. Generally, the TLV units are in mg × m^−3^ for substances that can exist as gas at 273 K and atmospheric pressure conditions. The HTPE is calculated using Equation (2):

(2) $$HTPE=\frac{1}{TLV}$$

The third toxicological category corresponds to aquatic toxicity potential (ATP), which is estimated using the LC_50_ index. LC_50_ represents the concentration of a substance that is lethal to 50% of a test population of *Pimephales promelas* (fathead minnows) within 96 h of exposure [23]. This species is used because it serves as a universal aquatic indicator and there are reported data concerning this species; it is quantified with Equation (3):

(3) $$ATP=\frac{1}{{LC}_{50}}$$

The fourth toxicological category is the terrestrial toxicity potential (TTP); it is estimated using the rat-oral value, ${LD}_{50}$, as shown by Equation (4) and has the same values as the HTPI.

(4) $$TTP=\frac{1}{{LD}_{50}}$$

The atmospheric categories are divided into four indicators, two of which are global, and the others local. The first of them, the global warming potential (GWP), is determined by comparing the extent to which a unit mass of a chemical absorbs infrared radiation over its atmospheric lifetime to the extent that CO_2_ absorbs infrared radiation over its respective lifetimes [23], this category is calculated by Equation (5):

(5) $$GWP=\frac{\int_{0}^{t}a_{i}c_{i}\left(t\right)dt}{\int_{0}^{t}a_{{CO}_{2}}c_{{CO}_{2}}\left(t\right)dt}m_{i}$$

where $a_{i}$ and $a_{{\mathrm{CO}}_{2}}$ are the radiation heat absorption per unit of greenhouse gas *i* and per unit of carbon dioxide; $c_{i}\left(t\right)$ and $c_{{\mathrm{CO}}_{2}}\left(t\right)$ are the concentration of a greenhouse gas i and the concentration of carbon dioxide in a time t after being released; t is the number of years (100) over which GWP is evaluated; and $m_{i}$ is the mass in kilograms of the emitted gas. The ozone depletion potential (ODP) considers the decomposition of chemical substances in the atmosphere, and it is quantified by comparing the reaction rate of a specific mass unit of a chemical with ozone to produce molecular oxygen with the reaction rate of the same mass unit of CFC-11 (trichlorofluoromethane) reacts with ozone to form molecular oxygen [23], and it is calculated by Equation (6):

(6) $$ODP=\frac{\delta {\left[O_{3}\right]}_{i}}{\delta \left[O_{3}\right]FCKW-11}m_{i}$$

where $\delta {\left[O_{3}\right]}_{i}$ is the global ozone depletion of a unit of gas i, and $\delta [O_{3}]\mathrm{FCKW}\text{-}11$ is the global ozone depletion of a unit of gas CFC-11, and $m_{i}$ is the mass in kilograms of an emitted gas.

The photochemical oxidation potential (PCOP) is quantified by comparing the rate at which a unit mass of a chemical reacts with a hydroxyl radical (OH^-^) to the rate at which a unit mass of ethylene has the same reaction [23]. This indicator focuses on low and medium-weight hydrocarbons, and it is calculated by Equation (7):

(7) $$PCOP=\frac{\frac{a_{i}}{b_{i}(t)}}{\frac{a_{C_{2}H_{4}}}{b_{C_{2}H_{4}}(t)}}m_{i}$$

where $a_{i}$ and $a_{C_{2}H_{4}}$ are the change in ozone concentration due to the change in a volatile organic compound *i* emission and due to the change in ethylene emission; $b_{i}\left(t\right)$ and $b_{C_{2}H_{4}}\left(t\right)$ is the integrated emission of a volatile organic compound *i* up to a time *t* and the integrated emission of ethylene at the same time; and $m_{i}$ is the mass in kilograms of the volatile organic compound emitted. 

Acidification potential or acid rain potential (AP) is determined by comparing the rate of release of H^+^ in the atmosphere as promoted by a chemical to the rate of release of H^+^ in the atmosphere as promoted by SO_2_ [23], and it is calculated with the Equation (8):

(8) $$AP=\frac{\frac{V_{i}}{M_{i}}}{\frac{V_{{SO}_{2}}}{M_{{SO}_{2}}}}m_{i}$$

where $V_{i}$ and $V_{{\mathrm{SO}}_{2}}$ are the acidification process of component i and SO_2_; $M_{i}$ and $M_{{\mathrm{SO}}_{2}}$ are the mass unit of components i and SO_2_; and $m_{i}$ is the mass in kilograms of the component i emitted.

The WAR algorithm establishes a connection between PEI (potential environmental impact) and the movement of an environmental impact that moves through a system’s boundaries, which occurs due to the exchange of mass and energy across those exact boundaries (PEI balance) [24]. The algorithm deals with two categories of indicators to assess the environmental impact of a chemical process. The first type measures the PEI emitted (output) by the process and the second type measures the PEI generated. The main aim of the output PEI is to evaluate the process’s external environmental effectiveness, which is the process’s ability to produce end products with minimum PEI discharge. As for the PEI, generation is used to understand the process’s internal environmental efficiency. Each category includes two indices: the potential impact per unit of time and per unit of product mass. The PEI per unit of product mass enables the comparison of processes and products based on the quantity of potential new environmental impacts generated. On the other hand, the PEI per unit of time is a valuable indicator to compare processes based on their rate of impact generation. The total output rate of PEI is calculated with Equation (9); the total mass output rate of PEI with Equation (10); The total generated rate of PEI by Equation (11); and the total mass generation rate of PEI with Equation (12).

(9) $$i_{out}^{\left(t\right)}=i_{out}^{\left(cp\right)}+i_{out}^{\left(ep\right)}+i_{we}^{\left(cp\right)}+i_{we}^{\left(ep\right)}=\sum_{j}^{cp}{M_{j}}^{\left(out\right)}\sum_{k}^{cp}X_{kj}\psi_{k}+\sum_{j}^{ep-g}{M_{j}}^{\left(out\right)}\sum_{k}^{ep-g}X_{kj}\psi_{k}$$

(10) $$i_{out}^{\left(t\right)}=\frac{\left(i_{out}^{\left(cp\right)}+i_{out}^{\left(ep\right)}+i_{we}^{\left(cp\right)}+i_{we}^{\left(ep\right)}\right)}{\sum_{P}P_{P}}=\frac{\sum_{j}^{cp}{M_{j}}^{\left(out\right)}\sum_{k}^{cp}X_{kj}\psi_{k}+\sum_{j}^{ep-g}{M_{j}}^{\left(out\right)}\sum_{k}^{ep-g}X_{kj}\psi_{k}}{\sum_{P}P_{P}}$$

(11) $$\begin{array}{l} i_{gen}^{\left(t\right)}=i_{out}^{\left(cp\right)}-i_{in}^{\left(cp\right)}+i_{out}^{\left(ep\right)}-i_{in}^{\left(ep\right)}+i_{we}^{\left(cp\right)}+i_{we}^{\left(ep\right)}\\ \;\;\;\;\;\;=\sum_{j}^{cp}{M_{j}}^{\left(out\right)}\sum_{k}^{cp}X_{kj}\psi_{k}-\sum_{j}^{cp}{M_{j}}^{\left(in\right)}\sum_{k}^{cp}X_{kj}\psi_{k}+\sum_{j}^{ep-g}{M_{j}}^{\left(out\right)}\sum_{k}^{ep-g}X_{kj}\psi_{k} \end{array}$$

(12) $$\begin{array}{l} i_{gen}^{\left(t\right)}=\frac{\left(i_{out}^{\left(cp\right)}-i_{in}^{\left(cp\right)}+i_{out}^{\left(ep\right)}-i_{in}^{\left(ep\right)}+i_{we}^{\left(cp\right)}+i_{we}^{\left(ep\right)}\right)}{\sum_{P}P_{P}}\\ \;\;\;\;\;\;=\frac{\left(\sum_{j}^{cp}{M_{j}}^{\left(out\right)}\sum_{k}^{cp}X_{kj}\psi_{k}-\sum_{j}^{cp}{M_{j}}^{\left(in\right)}\sum_{k}^{cp}X_{kj}\psi_{k}+\sum_{j}^{ep-g}{M_{j}}^{\left(out\right)}\sum_{k}^{ep-g}X_{kj}\psi_{k}\right)}{\sum_{P}P_{P}} \end{array}$$

where ${i_{out}}^{\left(cp\right)}$ and ${i_{in}}^{\left(cp\right)}$ are the rate of PEIs out and into the system attributable to chemical interactions inside the system, respectively; ${i_{out}}^{\left(ep\right)}$ and ${i_{in}}^{\left(ep\right)}$ are the rates of PEI out and into the system because of the energy generation processes within the system; ${i_{we}}^{\left(ep\right)}$ and ${i_{we}}^{\left(cp\right)}$ are the PEIs out of a system as a result of the waste energy released due to energy generation and chemical processes inside the system. ${M_{j}}^{\left(in\right)}$ and ${M_{j}}^{\left(out\right)}$ are the input and output mass flow rates of stream *j*, $X_{k}$ is the mass fraction of a component *k* in the stream *j*, $\psi_{k}$ is the overall potential environmental impact of chemical *k*, and $P_{p}$ is the mass flow rate of product p [25].

### 2.2. Process Description

**Figure 1:**
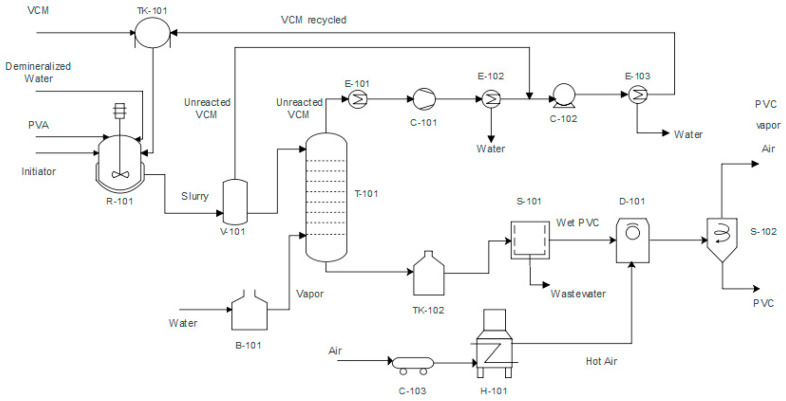
Process Flow diagram of PVC suspension production process.

Figure 1 shows the process flowsheet of the PVC suspension process that consists of a VCM polymerization reaction stage, PVC purification stage, unreacted monomer recovery stage, and PVC drying stage (water removal). This topology is constructed from actual chemical plant data and scientific literature [26,27,28]. For the polymerization reaction, a liquid VCM stream (fresh and recirculated) at atmospheric temperature and a pressure of 4.5 kg-f × cm^−2^ enters a reactor (R-101) at a temperature of 70 °C and a pressure of 10 kg-f × cm^−2^, together with demineralized water at 3.5 kg-f × cm^−2^ and 85 °C, a 20% dissolved polyvinyl alcohol (PVA), and 3-Hydroxy-1,1-dimethylbutane-2 ethyl-2-methylheptane peroxide (as initiator) dissolved at 20%, both at 10 kg-f × cm^−2^ and 32 °C. The polymer is produced (with conversion around 85%) within the droplets of the monomer in a suspension of water at a constant temperature and agitation. This reaction is exothermic, so excess energy is removed. The generated polymer creates a new phase as it is not soluble in the monomer and forms particles of different sizes. At the end of the reaction, a heterogeneous mixture called “slurry” remains, which contains both the suspended polymer, the unreacted monomer, water, the initiator, and the stabilizer. This mixture is at a pressure of 3.5 kg-f × cm^−2^ and a temperature of around 70 °C. The slurry stream leaving the reactor has a high content of residual monomer that must be removed due to its high toxicity. According to international regulations, the VCM content in the polymer should not exceed 1 ppm.

A flash separator (V-101) is used for the gasification stage to purify the monomer, where the pressure is reduced to 1.8 kg-f × cm^−2^. This pressure change separates the unreacted, volatile monomer from the liquid phase of the suspension (around 95%). Subsequently, the resulting liquid stream retains a fraction of the monomer (5%) that must be removed. Therefore, the stream enters the stripping column (T-101), which consists of a trayed tower (21 trays), where a stream of vapor generated in a high-pressure boiler (B-101) enters from the bottom with high temperature (225 °C) and pressure (14 kg-f × cm^−2^) and carries the monomer from the mixture that falls down the tower, resulting in a top stream rich in monomer and a bottom stream free of VCM with less than 1 ppm of the monomer.

The rich stream in unreacted monomer at the top enters the monomer recovery system, which consists of a series of compressors and heat exchangers that condition the residual VCM for recirculation (removal of water and turning the monomer into a liquid state). This stream first enters a heat exchanger (E-101) to be cooled to 50 °C, then passes through a vacuum pump (compressor, C-101) that allows the vapor to reach saturation point before entering a condenser (E-102) to separate the water from the VCM gas stream. Subsequently, the gas streams leaving the condenser and gasification stage enter a compressor (C-102). They are brought to a pressure of 3.5 kg-f × cm^−2^ to be conditioned close to the saturation pressure of the monomer for easy condensation through a heat exchanger (E-102) and to be recirculated to the process.

The bottom stream from the stripper with the monomer has a high water content (approximately 70%) that needs to be removed. The suspension enters a centrifuge (S-101) rotating at 1800 rpm, separating around 75% of the water. The residual water stream carries fractions of the polymer, almost all of the PVA, and the initiator. The product stream comes out of the centrifuge as a wet paste with moisture above the desired specification (below 10%). In the drying stage, the PVC paste is dried with an air stream at approximately 250 °C in a rotary dryer (D-101). The resulting moisture content of the polymer is 0.01% by weight [29].

The stream leaving the dryer is a gas mixture of air, steam, and polymer particles. To separate the dry polymer from the residual gas mixture, a cyclone (S-102) is used, which operates at atmospheric pressure (1.03 kg-f × cm^−2^). The particles’ inertia removes the solid polymer particles from the gas phase. The stream leaving the top of the cyclone is air and vapor along with a fraction of the polymer (0.2% of the total produced), and the bottom stream is a dry and granulated polymer with 0.01% water.

### 2.3. Environmental Assessment Using Computer-Aided Process Engineering

The environmental assessment of the suspension PVC production process was carried out using the WAR GUI^®^ software, which is based on the waste reduction algorithm (WAR). A workstation is used with an Intel processor (4 cores and 4.10 GHz), an Nvidia^®^ graphics card of 16 GB, 64 GB of memory, and a solid disk of 2 TB. 

**Figure 2:**
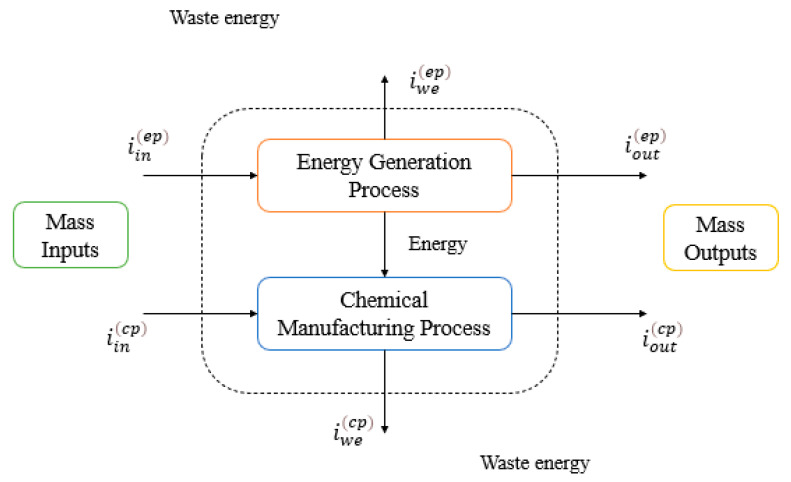
PEI balance for chemical processes considering energy consumption.

To quantify the global impacts, Equations (9)–(12) formulated from the PEI balance were used, as shown in Figure 2, considering the material flows moving within the process (inputs and outputs) and their energy consumption. For both atmospheric and toxicological categories, data from the streams, such as chemical composition and toxicological and atmospheric properties of the compounds, were used with Equations (1)–(8).

To visualize the values of the estimation total and by categories of impact indicators, bar charts were made, considering four cases: Case 1, also known as the baseline, in which only the impacts of waste are considered without accounting for the contribution of energy resources and product flows. Case 2 considers the impacts of product flow and waste without the contribution of energy. Case 3 considers the impacts of waste and the contribution of energy consumption. Case 4 considers the impacts of both product flow and energy consumption, as well as the impacts of waste. All four cases were used for the total impact chart, while for individual atmospheric and toxicological categories, only Case 4 was used. Additionally, impacts were quantified by category for the energy flow and energy source using Case 3, which provides a good diagnosis of the environmental performance of the process according to the contribution of energy consumption. In addition, the output rate of PEI per day was evaluated for the different substages (sections) of the process; only the wastes and energy contribution were considered.

## 3. Results and Discussion

**Figure 3:**
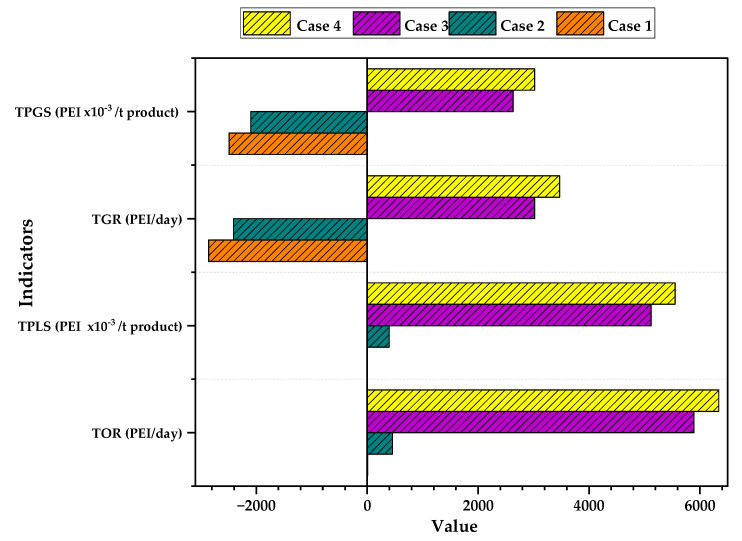
Total PEI generated and output of PVC suspension process.

Figure 3 shows the results of the total PEI generated and total output PEI per ton of PVC and per day for all the cases studied. It can be observed that for Case 1 and Case 2, the rate of PEI generation is negative (−2800 and −2400 PEI/day). These values indicate that the process consumes environmental impacts by transforming a substance with high environmental impacts such as VCM, which has an LD_50_ of 500 mg/kg, into a compound with lower potential global impacts such as PVC, which has an LD_50_ of 2000 mg/kg. On the other hand, the opposite happens for Cases 3 and 4, in which the generation of PEI per unit of time is positive (3020 for Case 3 and 3470 PEI/day for Case 4). The results of Cases 3 and 4 show that energy consumption has significant effects on the PEI generation of the process due to the emission of gaseous substances with high environmental impacts.

In Figure 3, it can be observed that Case 1 has the lowest value of output PEI with 6.5 PEI/day, which is due to the process not having any outputs of hazardous substances such as VCM (it is recirculated) in waste streams. For Case 2, a higher value of 457 PEI/day is presented, indicating that the product (PVC) has a significant contribution to the output PEI; the same happens with Case 3 and Case 4, with values of 5890 PEI/day and 6340 PEI/day respectively, showing that energy consumption significantly influences the increase in the rate of impact emissions in the process compared to products and waste. Comanita et al. confirm these statements by placing the impacts from energy consumption within the significant environmental impacts that exist in the PVC production chain [30].

The generation and output PEI per ton of product also had nominal values. Regarding the generated impacts, it is noteworthy that only Cases 3 and 4 showed values above 0 (3.02 and 5.5 PEI/t, respectively), unlike Cases 1 and 2, which were negative (−2.49 and −2.1 PEI/t). The difference between cases is associated with the effects of energy consumption. For the output PEI, all cases have positive but low values (between 0.005 and 5.51 PEI/t). This shows that the process produces PVC with minimum environmental impacts.

Environmental assessments in the literature are conducted for other polymer processes, such as the polypropylene (PP) process studied by Jimenez-Varon [31] and the polyethylene (PE) process analyzed by Velasquez-Barrios [32]. The global impact indicators such as output PEI per ton of product and per day can be used to compare each production process. The PP has an output PEI of 1200 PEI/t, much higher than the 5.51 PEI/t of the PVC process. This difference is particularly unexpected since the PVC process has a higher energy consumption of 4625 GJ/t, almost twice the energy consumption of the PP process, which is 2563 GJ/t although the capacity of the PP process is two times lower than the studied process. Additionally, the PP process has a propane output as a byproduct, which highly affects the formation of PEI (PCOP and HTPE).

On the other hand, the production process of PE has a higher emission rate than the suspension PVC production. The PE process has an output impact of 15,600,000 PEI/day, significantly higher (2461 times) than the 6340 PEI/day of the PVC process. The higher PEI rate of the PE process can be attributed to its low raw material conversion of 20% (a flow of 52 t/h of PE) unlike the suspension PVC process, which has conversions of 85%. In addition, the PE process does not recycle raw materials such as ethylene (which has considerable atmospheric and toxicological adverse effects) like the PVC process, which recirculates 99% of the VCM. Additionally, the presence of higher operating temperatures between 150 and 300 °C results in considerable energy consumption. The opposite happens for the PP process, which has temperatures of 80 °C, and the PVC process, which has temperatures between 70 and 250 °C. All this shows that the suspension PVC process has better environmental performance than other highly used polymers, thanks to the recycling of compounds derived from fossil fuels (VCM), high conversion, and medium operating temperatures (70–250 °C).

Table 1 shows an individual analysis of the process stages considering the waste contribution. The analysis was conducted to know the waste streams of each stage. The only stage with a significant output PEI is the drying stage with 6.5 PEI per day. This value is associated with the waste streams of the centrifuge and the dryer; both have a high content of substances with environmental impacts, such as VCM, PVA, the initiator, and PVC. On the other, the recovery stage has waste streams, but these streams are only water, which has no environmental impact, getting a value of 0. The reaction and purification stages have no streams that exist in the process as waste, only intermediary streams that cannot be assessed in the WAR algorithm. These values are not the same when energy is considered.

**Figure 4:**
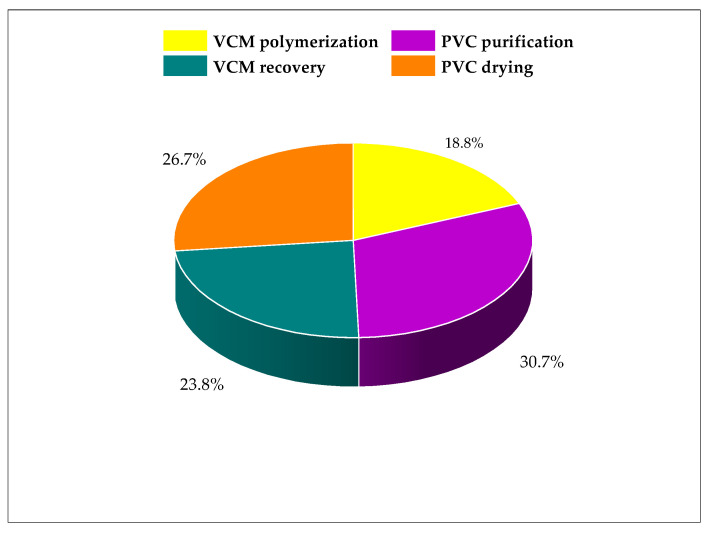
Contribution of output rate of PEI per process section.

Figure 4 shows the individual stages’ contribution to the PEI output of the process considering energy consumption. The PVC purification stage contributes the most to the total output of PEI with 31%. This stage has the highest temperature of the process, 250 °C, which incurs higher energy use. For the same reason, the drying stage contributes 27% of the total output. The PEI comes from the air heater at 225 °C. On the contrary, the VCM polymerization and VCM recovery stages make less contribution (24% and 18%, respectively) due to not involving the direct use of energy for heating purposes. These stages are categorized as using energy in the form of work and the use of refrigerant for cooling.

### 3.1. Toxicological Impacts of PVC Suspension Production Process

**Figure 5:**
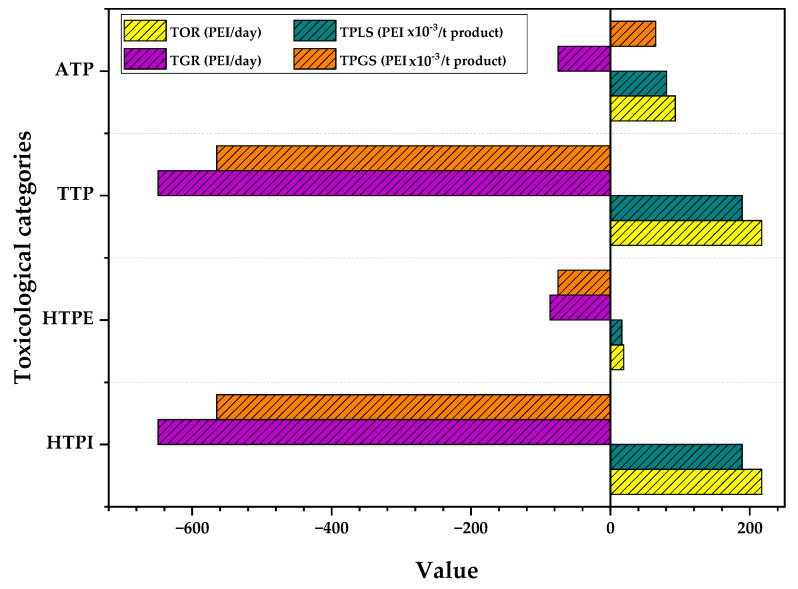
Toxicological impacts of the PVC suspension production process including waste streams, product streams, and energy consumption.

Figure 5 shows the rate of toxicological impacts generated and output from the suspension PVC production process, including impacts on humans (HTPI and HTPE) and aquatic and terrestrial resources (ATP and TTP). The categories HTPI, HTPE, and TTP have a negative rate of generated PEI (−649, −86.6, −649 PEI/day, respectively); these values indicate that the process takes raw materials such as VCM with higher PEI in those categories and converts them into products with lower impacts such as PVC.

On the contrary, the rate of PEI generated for the ATP category shows a positive value (74.9 PEI/day) due to the influence of compounds (initiator, PVA) that are released in waste streams (such as wastewater from the centrifuge) and the product stream (the stream that exits the cyclone bottom) with an LC_50_ of 200, 1, and 100 mg/L, respectively. Additionally, the emission of substances associated with energy requirements can contribute to the increase in the category value, such as low molecular weight VOCs that are easily absorbed by bodies of water [33].

The figure shows that all categories have appreciable values (above 0) for the output impacts. For this indicator, it is observed that the HTPI and TTP categories have the same value (217 PEI/day). These values are mainly related to the product stream, which has a higher flow (1150 t/day) than other substances that are discharged as waste, such as the initiator or PVA (0.3 and 1.7 t/day, respectively). The PVC almost entirely influences the value of the HTPE category since it is the only compound suspended in the air. For the HTPE category, only substances in a gaseous state or those that can remain in the air can contribute to the increase in PEI. Therefore, the output flows of PVC influence the impacts of this category. Although the polymer’s TLV (15 mg/m^3^) is significantly lower than the TLV of VCM (2 mg/m^3^), however, the VCM is completely recycled (does not get out of the process in high amounts). This same reason explains why the HTPE category has a higher generated PEI value (−87 PEI/day) than the HTPI and TTP categories (−649 for both).

The PEI generated per ton of product presents a positive value for the ATP category, contrary to what happens for the HTPI, HTPE, and TTP categories, where they have negative values. This value is due to not only the contribution of waste and product streams but also gases emitted by energy consumption. On the other hand, the output impacts of the process per mass unit show that all the categories present values lower than 1 (0.189, 0.0165, 0.189, 0.0805 for HPTI, HPTE, TTO, ATP, respectively), and these categories are influenced mainly by the product and its low toxicological effects.

### 3.2. Atmospheric Impacts of PVC Suspension Production Process

**Figure 6:**
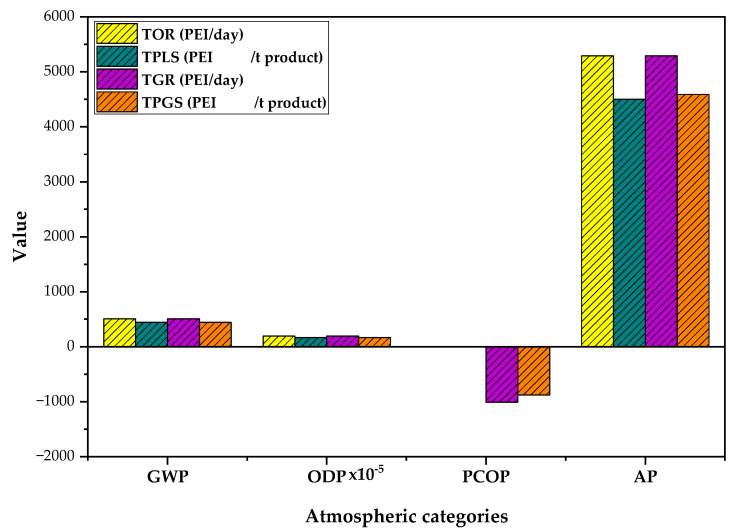
Atmospheric impacts of the PVC suspension production process.

Figure 6 shows the global (GWP and ODP) and local (PCOP and AP) atmospheric impacts for the PVC suspension production process. For the ODP and PCOP categories, the PEI generated (0.0001 and −1,000 PEI/day, respectively) and output (0.0001 and 0.3 PEI/day, respectively) values are less than 1 PEI/day. There are no substances released in the waste or product streams that contribute to these impacts, due to them being mainly liquid or solid. The only gaseous stream that exits the process is the waste stream from the top of the cyclone (mixture of air and PVC), but this stream contributes mainly to toxicological impacts. Additionally, the VCM can emit impacts related to the PCOP category. However, this process recirculates all unreacted VCM flows. Therefore, the topology does not present significant output flow (less than 1 ppm) that can significantly influence the value of this category. So, the value presented is due to low molecular weight organic compounds such as methane and VOCs related to energy consumption.

The GWP and AP categories present appreciable values for the PEI generated and output indicators in comparison with the other atmospheric and toxicological categories due to the emission of different chemical species in the vapor phase as waste from energy consumption. For the GWP category, it showed values of 510 PEI/day for both indicators (output and generated). These values are mainly due to the emission of carbon oxides (CO_x_) generated by the burning of fossil fuel, which for this study was defined as natural gas taking into account the actual operating conditions of PVC plants [34]. Likewise, the AP category is influenced by the release of gases such as VOCs and NO_x_ (for natural gas), with a value of 5290 PEI/day for both indices [35]. The AP category represents 83% of the output impacts and 89% of the generated impacts, showing that the process’s energy consumption has a high contribution to the impacts generated by the process related to the atmospheric impacts.

On the other hand, the PEI per ton of product shows that the process has low impacts concerning the flow of PVC produced; this indicates that the process can produce the PVC in an environmentally friendly manner since the values do not exceed the 1 PEI/t. Furthermore, the high product flow (1152 t/day) allows the process to generate the polymer with less environmental impact.

### 3.3. Impacts According to the Energy Source Used by the Process

**Figure 7:**
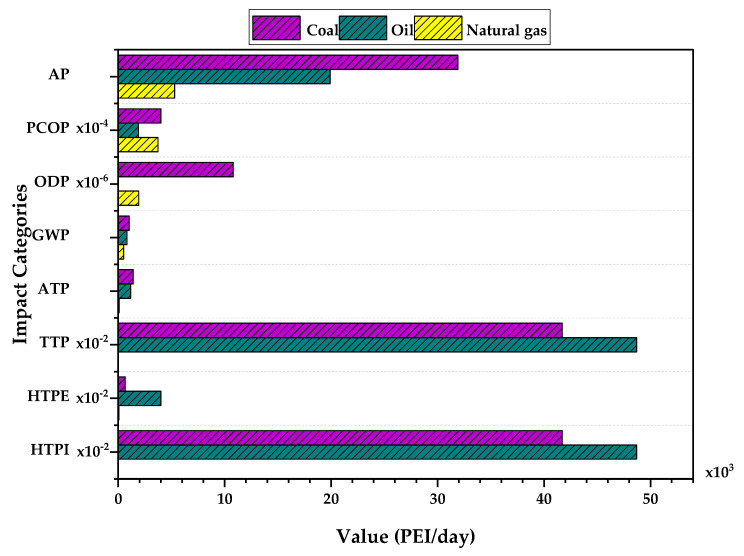
Output rate of PEI from energy usage comparison for PVC suspension production process.

Figure 7 shows the PEIs for the atmospheric and toxicological categories according to the type of fuel used to satisfy the energy requirements. For this analysis, Case 4 was taken into account, which considers the contribution of the waste, product, and energy used to obtain the PVC. The AP category is most affected by energy consumption for the three energy sources (between 5000–35,000 PEI/day), due to the emission of gases such as NO_x_ and SO_x_, which can cause acidification by reacting with atmospheric water vapor [36]. As expected, coal presents the worst performance with an acidification potential almost double that of liquid fuels and six times greater than natural gas. The chemical structure of coal has a higher content of compounds such as sulfides, nitrogen, and volatile compounds [37] compared to gaseous and liquid fuels. In addition, it is observed that in the other categories of atmospheric impacts, only GWP has significant values (between 500–1000 PEI/day); the discharge of carbon oxides (CO_x_) by combustion influences the increase in this category. On the contrary, the PCOP and ODP categories have nominal emission rates of impact (less than 1 PEI/day).

The output PEIs of the toxicological categories show an appreciable contribution from energy consumption. The ATP category presents the most significant impacts due to the generation of VOCs such as benzene and polycyclic aromatic hydrocarbons (PAHs) that tend to stay in bodies of water because of their ubiquity. The other toxicological categories such as TTP, HTPE, and HTPI present slightly lower impacts in comparison; however, they are influenced by the ATP category, by volatile gases such as naphthalene and particulate matter. Additionally, it can be seen from the figure that natural gas presents better performance than the other fuels, so it is advisable to use it as the primary fuel to meet the energy needs of the process.

## 4. Conclusions

The waste reduction algorithm (WAR) was implemented to evaluate the environmental performance of the PVC production process by suspension with unconverted monomer recovery and resin drying. The process transforms raw materials with high PEI such as VCM into final products with lower PEI such as PVC; that means that PEI is consumed. On the other hand, the process presents a high generation of PEI because of the energy consumption; this is reflected in the fact that the total generated PEI presented positive values in two of the four cases. The evaluation of the toxicological impacts shows that the process performs well since only the ATP category presents a positive PEI generation value; the opposite occurs for the other toxicological categories (HTPI, HTPE, and TTP). On the other hand, it was determined that the product contributes to the formation and emission of PEI. In addition, the categories analyzed showed that the energy consumption of the process is the most critical aspect under environmental criteria since the impacts derived from energy consumption represented 91% of the total impacts emitted. The AP and GWP categories presented the highest values and are directly associated with greenhouse gas emissions. In addition, it was determined that the most convenient fuel is natural gas due to its lower impacts than liquid and solid fuels (coal). At the same time, it can be concluded that the production process of PVC by suspension is environmentally acceptable compared with other polymers such as polyethylene or polypropylene due to its lower output impacts. Finally, the application of exergy analysis is recommended to evaluate the efficient use of energy in the process with energy integration to reduce fuel use.

## Figures and Tables

**Table 1:** Stages’ contribution to the total output rate of PEI.

Stage	Output Rate of PEI Per Day	Contribution (%)
VCM polymerization	0	0%
PVC purification	0	0%
VCM recovery	0	0%
PVC drying	6.5	100%
total	6.5	100%

## Data Availability

The data that support the findings of this study are available from the corresponding author, Á.D.G.-D., upon reasonable request.

## Nomenclature

PEI	Potential environmental impact
PVC	Poly(vinyl chloride)
PE	Polyethylene
PP	Polypropylene
WAR	Waste reduction algorithm
AP	Acidification potential
GWP	Global warming potential
PCOP	Photochemical oxidation potential
ODP	Ozone depletion potential
HTPI	Human toxicity potential by ingestion
HTPE	Human toxicity potential by exposition
TTP	Terrestrial toxicity potential
ATP	Aquatic toxicity potential
ORE	Output rate of PEI from energy usage (PEI/h)
PAH	Polycyclic aromatic hydrocarbon
TGR	Total generation rate of PEI (PEI/day)
TOR	Total output rate of PEI (PEI/day)
TPGS	Total PEI generated within a system per mass of products (PEI/t product)
TPLS	Total PEI leaving the system per mass of products (PEI/t product)
VOC	Volatile organic compounds
VCM	Vinyl chloride monomer
OSHA	Occupational Safety and Health Administration
ACGIH	American Conference of Governmental Industrial Hygienists
NIOSH	National Institute for Occupational Safety and Health
